# Impairments and comorbidities in adults with cerebral palsy and spina bifida: a meta-analysis

**DOI:** 10.3389/fneur.2023.1122061

**Published:** 2023-07-18

**Authors:** Jane N. T. Sattoe, Sander R. Hilberink

**Affiliations:** Research Center Innovations in Care, Rotterdam University of Applied Sciences, Rotterdam, Netherlands

**Keywords:** cerebral palsy, spina bifida, muscular disease, comorbidity, impairment, prevalence, meta-analysis, epidemiology

## Abstract

**Introduction:**

Aging with a childhood-onset disability, such as cerebral palsy (CP), spina bifida (SB), and muscular diseases (MD), comes along with significant impairments and comorbidities. Despite the increasing evidence an overall picture is lacking. This study aimed to review the literature about adults with CP/SB/MD and impairments and comorbidities to perform a meta-analysis.

**Materials and methods:**

Embase, PubMed, Cinahl, and Google Scholar were searched (2000–2020). Search terms included adults with one of the aforementioned disabilities combined with impairments and comorbidities. If specific impairments or comorbidities were reported by at least four studies, these were included in the study. Pooled prevalence (95% Confidence Interval) of impairments/comorbidities were calculated.

**Results:**

The search yielded 7,054 studies of which 95 were included in the meta-analysis (64 CP, 31 SB, 0 MD). In total estimates were calculated for 26 (CP) and 11 (SB) outcomes. In adults with CP, pain [56.4% (95%CI 48.8–63.8)], deformities [44.2% (95%CI 12.9–78.4)], intellectual disability [37.2% (95%CI 26.7–48.3)], and fatigue [36.9% (95%CI 24.6–50.1)] were most prevalent; renal disease [3.0% (95%CI 2.1–4.2)] and stroke/rheumatic diseases {4.8% (95%CI 3.4–6.5; 4.8% (95%CI 1.5–9.9)] respectively} were least prevalent. For adults with SB, bladder incontinence [60.0% (95%CI 50.5–69.2)], bowel incontinence [49.2% (95%CI 34.5–64.0)], pain [44.1% (95%CI 27.4–61.5)], and sleeping problems [30.3% (95%CI 4.7–65.8)] were most prevalent; diabetes [4.8% (95%CI 2.8–7.3)] and renal disease [8.7% (95%CI 2.0–19.9)] were least prevalent. The included studies showed large heterogeneity.

**Conclusions:**

More research is needed to study health issues in adults with MD. Adults with CP or SB deal with a variety of health issues. More attention for the mental health of these adults is needed. There also is a need for accessible and adequate screening, preventive measures and clinical follow-up.

## 1. Introduction

Healthcare for adults with life-long disabilities has gained attention in the literature in the last two decades. Ample research showed increased impairments and comorbidities (also referred to as health issues) in these adults as they age (1–3). Many studies target specific adult populations, such as cerebral palsy (CP), spina bifida (SB) or muscular diseases (MD) [i.e., spinal muscular disease (SMA) or Duchenne muscular disease (DMD)/Becker muscular disease (BMD)]. Of these, adults with CP have been studied most (4–10).

Pain, fatigue, epilepsy and asthma are prevalent in adults with CP (11). In addition, these adults are at risk of several health complaints, including hypertension, depressive symptoms, osteoarthritis, cardiovascular diseases, type 2 diabetes (6, 12, 13). Adults with SB often experience bladder and bowel problems and fatigue (14, 15); fecal incontinence is more often observed with increasing age. Moreover these adults are at risk of renal failure (16). Adult men with DMD/BMD report urine incontinence (17), as well as psychiatric problems such as depressive and stress symptoms (18) and cardiac and renal dysfunction (19). Pain and fatigue are also common (20).

Recently three systematic reviews were published on adults with CP (11, 21, 22). These studies focused on specific health issues (pain and hypertension) or aimed at the most studied outcomes (including participation). As shown, adults with SB or MD develop significant health issues. However, they experience many barriers to healthcare services and screening (23, 24), hampering timely detection and secondary preventive measures. To inform both healthcare professionals as well as adults with CP, SB, or MD, we aim to estimate the prevalence of a broader scope of impairments and comorbidities in these adults. The present study goes beyond focusing on one diagnosis group and had more strict criteria to include outcomes to have more robust estimates. As such, it provides a broader overview of comorbidities that people with CP, SB, or MD often have to deal with than current literature does.

## 2. Methods

### 2.1. Study design and participants

We conducted a systematic review of the literature including meta-analysis to estimate the prevalence of impairments and comorbidities in adults with CP, SB, or MD. No review protocol was prepared, and the review was not registered in any register.

### 2.2. Search strategy

A search strategy was formulated and used in four databases: Embase, Pubmed, Cinahl, and Google Scholar. Search terms included the conditions “cerebral palsy”, “spina bifida”, “spinal muscular atrophy”, and “Duchenne muscular dystrophy” in combination with possible impairments and comorbidities such as “fatigue”, “pain”, and “diabetes. Some impairments and comorbidities were not included as search terms, but were still picked up, because they were often included as one among other outcomes in studies. This was the case for osteoporosis, obesity (as reflected by BMI) and gastroenterological problems. The full search strategy for Pubmed is presented in Supplementary material 1. After removing duplicates, publications were screened on title and abstract to check for eligibility (by both reviewers). Subsequently, full texts were screened, and disagreements were discussed and resolved.

### 2.3. Selection criteria

Studies were included if they met the following criteria:

1. Published in the period January 1^st^ 2000–December 31^th^ 2020;
2. Including a study sample of *n* ≥ 25;
3. All participants ≥18 years of age;
4. Not a follow-up intervention study;
5. No selected samples (i.e., only dyskinetic CP).

Of longitudinal designs, the most recent follow-up study with the specific comorbidity or impairment as outcome was included.

### 2.4. Data extraction

Data extraction was done by both reviewers with a standardized data extraction form in Microsoft Excel. Study sample characteristics and number of cases reported to have an impairment or a comorbidity were recorded for every study. Sample characteristics included first author and publication year, country, sex, age (mean/median), sample size and for CP the GMFCS levels (25). All impairments or comorbidities reported in the studies were recorded, but if less than four studies reported on a specific impairment or comorbidity, we did not include this outcome in the analysis.

### 2.5. Data analysis

Overall mean proportions and 95% Confidence Interval (95%CI) were estimated for each comorbidity. Random-effects meta-analysis models were used (with DerSimonian and Laird estimator). Meta-analysis modeling was done using the proportion meta-analysis function in StatsDirect. The random-effects model takes the heterogeneity of samples into account. The I^2^ measure indicated heterogeneity and represents the variation attributed to heterogeneity rather than sampling error across samples.

## 3. Results

### 3.1. Study characteristics

**Figure 1:**
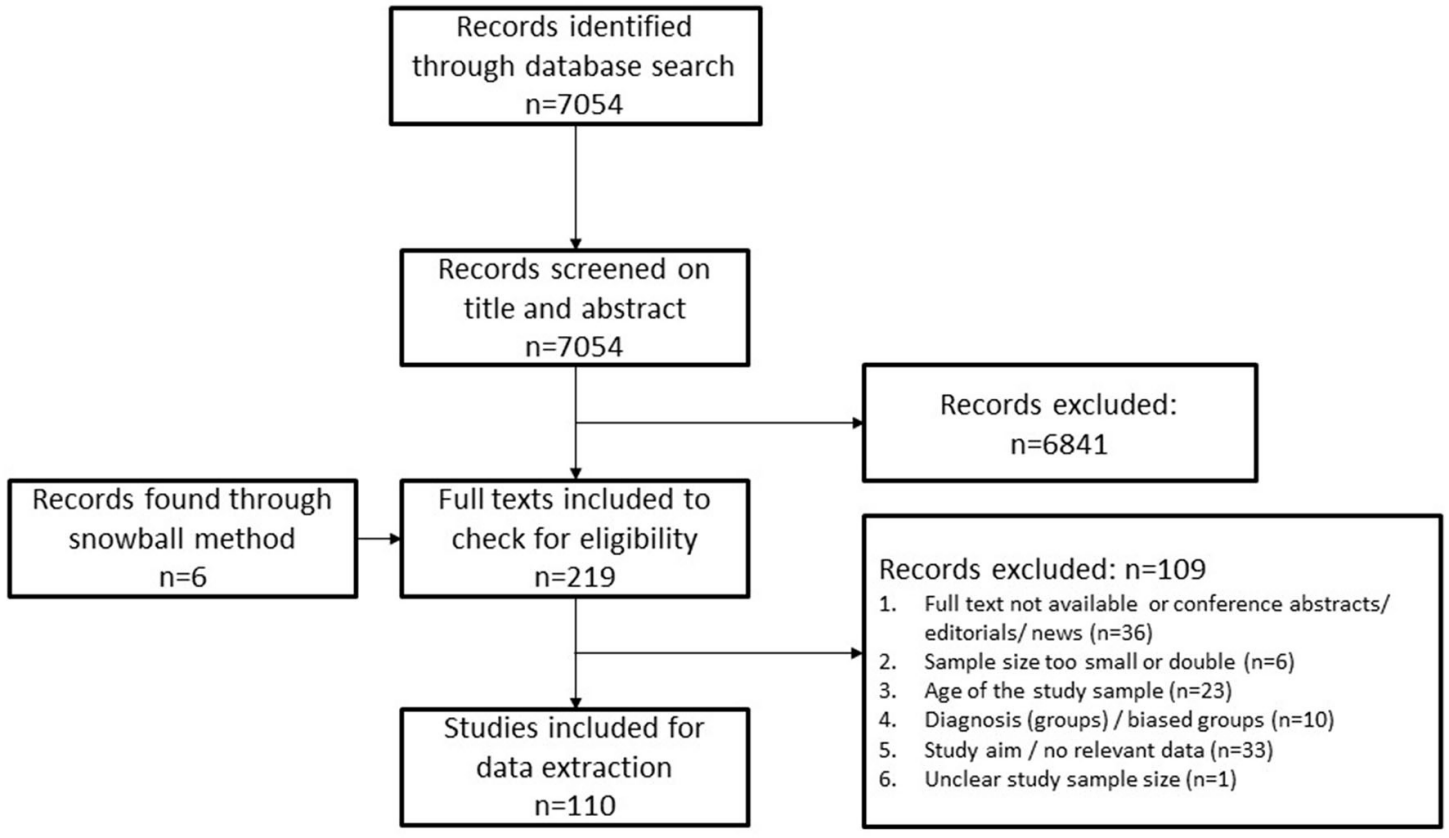
Search and selection of studies.

The full selection process is presented in Figure 1. In total 110 (of 7,054) studies met the inclusion criteria. Of these, 15 were excluded because these studies reported on impairments or comorbidities that were studied in less than three other studies. Of the 95 included studies, 64 reported on CP and 31 reported on SB. Regarding adults with MD, no outcome was reported more than three times, and therefore no results on health issues of these adults could be described. Risk of bias was assessed with a quality assessment checklist for prevalence studies that Nguyen and colleagues (26) adapted from Hoy and colleagues (27) and is presented in Table 1.

**Table 1:** Study characteristics and risk of bias assessment (*n* = 95).

**First author**	**Publication Year**	**Reference number**	**Country**	**Diagnosis**	**Sample N**	**Male, N**	**Mean/Median Age/Range (in years)**	**GMFCS I-III, N**	Risk of bias assessment score^*^
Andersson	2001	(28)	Sweden	CP	221	125	36	Not reported	2
Bellin	2013	(29)	USA	SB	48	22	22	-	2
Bendt	2020	(30)	Sweden	SB	196	92	33	-	2
Benner	2017	(31)	Netherlands	CP	49	27	40	39	3
Bottos	2001	(32)	Italy	CP	72	43	33	Not reported	2
Bowen	2021	(33)	USA	SB	75	34	22	-	2
Bowman	2001	(34)	USA	SB	71	33	22	-	1
Brochard	2017	(35)	France	SB	228	92	35	-	1
Chu	2019	(36)	USA	SB	75	34	20	-	1
Coco	2018	(37)	USA	SB	54	23	30	-	2
Cremer	2017	(38)	USA	CP	435	201	49	236	1
de la Torre-Olivares	2018	(39)	Spain	CP	30	14	31	30	4
Dicianno	2015	(40)	USA	SB	190	87	34	-	1
Dosa	2009	(41)	USA	SB	94	48	Range: 20–58	-	1
Edwards	2003	(42)	UK	SB	42	14	30	-	2
Ehrén	2020a	(43)	Sweden	SB	154	74	35	-	2
Ehrén	2020b	(44)	Sweden	SB	196	92	35	-	2
Engel	2003	(45)	USA	CP	100	55	41	18	1
Etter	2020	(46)	USA	CP	11,094	5,759	Not reported for whole study group	Not reported	0
Fortuna	2018	(47)	USA	CP	229	135	Not reported for whole study group	Not reported	1
Fowler	2015	(48)	USA	CP	48	21	34	26	1
French	2019	(49)	USA	CP	7,348	3,733	49	Not reported	0
Heyn	2019	(50)	USA	CP	70	34	25	70	3
Hilberink	2007	(51)	Netherlands	CP	54	26	30	37	3
Hirsh	2010	(52)	USA	CP	83	37	40	Not clear	2
Hung	2020	(53)	USA	CP	424	199	33	254	2
Içagasioglu	2020	(54)	Turkey	CP	70	37	29	40	3
Jacobson	2020	(55)	Sweden	CP	61	34	21	40	3
Jahnsen	2004	(56)	Norway	CP	406	209	34	Not reported	2
Jarl	2019	(57)	Sweden	CP	408	219	27	326	0
Jeon	2019	(58)	Korea	CP	80	46	43	37	3
Jonsson	2019	(59)	Sweden	CP	581	337	Range: 39–58	481	0
Liu	2016a	(60)	USA	SB	33	0	33	-	2
Liu	2015	(61)	USA	SB	66	22	32	-	2
Liu	2016b	(62)	USA	SB	225	95	30	-	1
Lundberg Larsen	2020	(63)	Norway	SB	26	10	Range: 51–76	-	3
Lundh	2018	(64)	Sweden	CP	50	26	32	50	3
Marciniak	2014	(65)	USA	CP	91	46	36	34	3
Marciniak	2015	(66)	USA	CP	91	46	36	34	3
McDermott	2005	(67)	USA	CP	177	83	32	Not reported	0
McDonnell	2000	(68)	UK	SB	193	95	28	-	1
McMorris	2021	(69)	Canada	CP	14,155	7,052	Range: 18–64	Not reported	0
McPhee	2015	(70)	Canada	CP	42	21	34	24	2
McPhee	2017	(71)	Canada	CP	41	20	34	24	2
Mezaal	2009	(72)	Iraq	CP	50	50	21	Not reported	2
Morley	2020	(73)	USA	SB	852	221	37	-	2
Nieuwenhuijsen	2011	(74)	The Netherlands	CP	42	29	36	42	0
Oakeshott	2003	(75)	UK	SB	57	25	30	-	1
Oakeshott	2007	(76)	UK	SB	50	Not reported	38	-	1
O'Connell	2019	(77)	UK	CP	1,705	907	Median: 29	Not reported	0
Opheim	2011	(78)	Norway	CP	149	76	40	127	0
Opheim	2009	(79)	Norway	CP	149	76	40	127	2
Park	2018	(80)	Korea	CP	154	93	40	79	2
Park	2017	(81)	Korea	CP	52	33	31	33 (without GMFCS III)	1
Peterson	2014	(82)	USA	CP	112	52	34	58	1
Peterson	2019	(10)	USA	CP	2,659	1,374	36	Not reported	1
Peterson	2012	(83)	USA	CP	43	23	37	29	3
Peterson	2015	(84)	USA	CP	1,015	669	58	Not reported	0
Pons	2017	(85)	France	CP	282	161	38	112	2
Roach	2011	(86)	USA	SB	84	Not reported	31	-	4
Rodby-Bousquet	2013	(87)	Sweden	CP	102	63	Median: 21 Range: 19–23	72	1
Ryan	2014	(88)	Ireland	CP	55	31	38	41	1
Ryan	2019	(89)	UK	CP	1,705	907	Median: 29	Not reported	0
Sandström	2004	(90)	Sweden	CP	48	23	33	34	1
Showen	2021	(91)	USA	SB	195	49	40	-	4
Sienko	2018	(92)	USA	CP	97	47	24	63	3
Slaman	2013	(93)	Netherlands	CP	36	23	36	36	1
Smith	2019	(94)	UK	CP	1,705	907	33	Not reported	0
Smith	2021	(95)	UK	CP	1,703	906	33	Not reported	0
Stepanczuk	2014	(96)	USA	SB	225	106	Not reported	-	1
Summers	2014	(97)	USA	SB	65	32	31	-	1
Trinh	2017	(98)	Australia	SB	49	20	Median: 33	-	1
Urrutia	2017	(99)	Chile	SB	235	95	38	-	1
Van der Slot	2012	(4)	Netherlands	CP	56	35	36	52	1
Van der Slot	2013	(100)	Netherlands	CP	43	27	36	41	1
Veenboer	2014	(14)	Netherlands	SB	61	22	Median: 45	-	2
Vukojevic	2017	(101)	Bosnia and Herzegovina	CP	100	62	Not reported	Not reported	2
Wagner	2015	(102)	USA	SB	72	25	Not reported	-	2
Werhagen	2013	(103)	Sweden	SB	127	61	34	-	1
Whitney	2018a	(104)	USA	CP	1,395	676	Not reported for whole study group	Not reported for whole study group	1
Whitney	2019a	(105)	USA	CP	5,052	50.4%	53	Not reported	1
Whitney	2020a	(106)	USA	CP	646	264	58	Not reported	1
Whitney	2020–2 = 2020b	(107)	USA	CP	9,357	4,820	Not reported for whole study group	Not reported	1
Whitney	2020–3 = 2020c	(108)	USA	CP	5,888	3,133	Not reported for whole study group	Not reported	1
Whitney	2020d	(109)	USA	CP	5,603	2,813	54	Not reported	1
Whitney	2021a	(110)	USA	CP	294	144	Not reported	158	3
Whitney	2021 = 2021b	(111)	USA	CP	9,238	4,635	49.5	Not reported	1
Whitney	2018b	(112)	USA	CP	452	43.4%	23.6	231	1
Whitney	2019–3 = 2019b	(6)	USA	CP	5,555	52.2%	42.3	Not reported	1
Whitney	2020e	(113)	USA	CP	8,011	4,012	49.4	Not reported	1
Whitney	2020f	(7)	USA	CP	17,212	9,213	Not reported	Not reported	1
Wiener	2017	(114)	USA	SB	1,370	582	Range: 20–83	-	0
Wiener	2018	(115)	USA	SB	1,372	583	Range: 20–83	-	0
Wu	2017	(116)	Taiwan	CP	1,975	911	Not reported	Not reported	0
Yildiz	2017	(117)	Turkey	CP	117	64	25	86	1

^*^Low risk: 0–3, moderate risk: 4–6, high risk: 7–9.

Almost half of all studies were conducted in the United States of America (USA) (ṇ = 47), followed by eleven studies from Sweden (12%), eight studies conducted in the United Kingdom (UK) (8%), and seven studies from The Netherlands (7%). Other studies were from different countries all over the world, but the number of studies for specific countries was small (range: 1–4). Sample size of the included studies varied from 26 to 17,212 people with CP or SB. One study only included females in the study population (Liu et al., 2016) and one study included only males (Mezaal et al., 2009). Other studies had mixed study populations in terms of sex, the proportion of males varied from 25 to 69%. Of these, 53 studies (57%) had a more or less equal distribution of males and females in the study population (between 45 and 55%). All study characteristics are presented in Table 1.

### 3.2. Health issues in adults with CP

**Figure 2:**
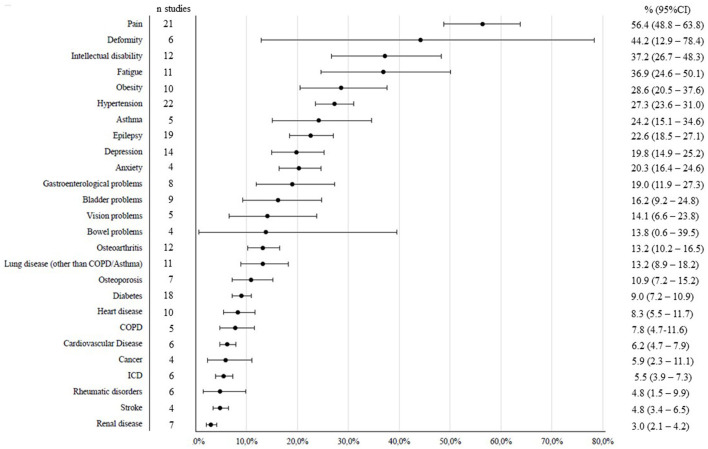
Impairments and comorbidities in adults with CP.

Figure 2 shows the number of studies per impairment or comorbidity and the estimated prevalence (95%CI) of these in adults with CP. A total overview of the health issues, the studies that reported on them, and the number of cases included in the analyses is given in Supplementary material 2. For all analyses the level of heterogeneity (I^2^) was high (>70%), indicating substantial variation in results across the studies.

The health issue that was most often included in study designs was hypertension (*n* = 22), followed by pain (*n* = 21) and epilepsy (*n* = 19). Least studied were stroke (*n* = 4), cancer (*n* = 4), bowel problems (*n* = 4), and anxiety (*n* = 4). The most prevalent comorbidity in adults with CP was pain, the overall prevalence was 56%. Deformities were the second most prevalent (44%). Intellectual disability, fatigue, obesity and hypertension, asthma, epilepsy, depression and anxiety were prevalent in more than 20% of adults with CP. Least common were renal diseases (3%), stroke (5%), and rheumatic disorders (5%).

### 3.3. Health issues in adults with SB

**Figure 3:**
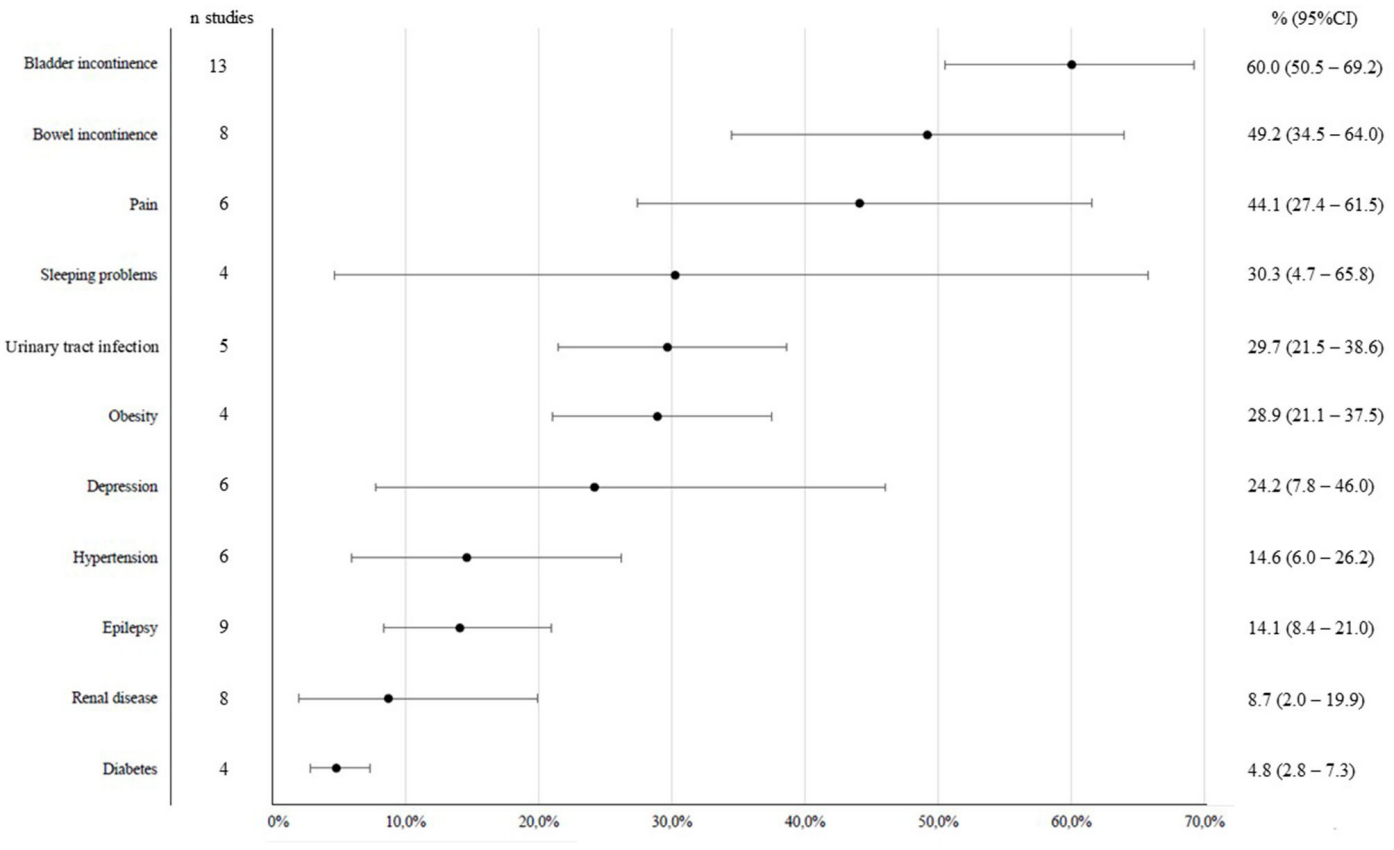
Impairments and comorbidities in adults with SB.

Figure 3 shows the number of studies per impairment or comorbidity and the overall proportion of these in adults with SB. A total overview of the health issues, the studies that reported on them and the number of cases included in the analyses is given in Supplementary material 3. For almost all of the analyses the level of heterogeneity was also high (>70%) except for diabetes (I^2^ = 41%).

The health issue that was most often included in study designs was bladder incontinence (*n* = 13), followed by epilepsy (*n* = 9) and renal disease (*n* = 8) and bowel incontinence (*n* = 8). Least studied were diabetes (*n* = 4), obesity (*n* = 4), and sleeping problems (*n* = 4). Bladder incontinence was most prevalent (60%), followed by bowel incontinence (49%) and pain (44%). Urinary tract infection, obesity and depression were present in more than 20% of adults with SB. Least common was diabetes (5%).

## 4. Discussion

### 4.1. Main findings

This meta-analysis is the first to review a wide scope of impairments and comorbidities in adults with CP or SB. Impairments and comorbidities in adults with MD could not be assessed due to limited studies. The overall picture may inform health professionals and adults with CP or SB about the common prevalent health issues.

For adults with CP, the results show a lower estimated prevalence of pain (56%) compared to a previous meta-analysis [70%; (21)]. This perhaps has to do with the difference in included studies and thus in other samples concerning sex, age, GMFCS levels, and subtypes. Nevertheless, pain is the most prevalent impairment reported, not only in our study but also in another recent study (11); it was also the second most studied health issue. Attention for pain as a health problem in adults with CP is important in clinical practice (118). This is also the case for adults with SB: They also have a high prevalence of pain (44%), but less often studied. Pain is found to be an important health issue for almost all people with childhood-onset disabilities, starting from a young age. Research also shows that pain can have a profound impact on quality of life and mental health (119).

There seems to be a lack of attention for mental health. While for both depressive symptoms and anxiety, the prevalence rates in adults with CP are around 20%, these outcomes belong to the less studied ones, and this is especially true for anxiety. On the contrary, epilepsy and hypertension are not much more prevalent, but far more often studied, with hypertension being the most studied outcome among adults with CP in this review. For adults with SB, the same pattern was found. While depressive symptoms were prevalent in almost 25% of the people with SB, these were studied in < 50% of the studies included. Other original studies also highlighted the risk of depression and anxiety in people with CP and SB and the need for more attention for mental health in these groups (94, 120, 121). Moreover, literature suggests that comprised mental health is associated with health issues such as pain and fatigue (4, 20). The results call for attention for mental health.

The overall results of this meta-analysis show profound health issues that people with CP and SB have to deal with. They have increased medical needs compared to the general population. Yet, screening of people with CP or SB on health issues is not common practice yet (122–124) and access to needed healthcare is not always self-evident (125). More attention is needed for this matter of how current healthcare practice can be tailored to these increased needs of people with CP or SB. The need for prevention and clinical follow-up of health issues (including mental health) has been emphasized before (120, 126). Moreover, comorbidities not only reflect medical challenges, preventive measures may positively impact social participation of adults with lifelong disabilities as well (125, 127).

### 4.2. Limitations

It is important to acknowledge the high levels of heterogeneity (I^2^) in our analyses, indicating substantial variation in results across studies. These levels show that there is no clear pattern of comorbidities or impairments across studies. Yet, we felt it appropriate to summarize the outcomes, because the level of heterogeneity can also be influenced by the fact that outcome measures were not measured in a uniform way across studies. Also, there are differences in sex, age, disability and subtypes of conditions in the study samples. A limitation of this study is also that, due to a small number of studies, other conditions than CP and SB (e.g., MD) could not be included in the analysis. Finally, it must be emphasized that most studies included in this meta-analysis were performed in high-income countries. Therefore, it is not representative for the whole world. More research in low- and middle-income countries is warranted.

### 4.3. Conclusions

Health issues in adults with MD are studied too less to perform a meta-analysis. Hence research on the impairments and comorbidities in this population is strongly recommended to inform health professionals and the adults themselves. Adults with CP or SB have to deal with a variety of health issues next to their main disability. Pain is found to be the most prevalent issue and can have profound impact on quality of life and mental health. Mental health of adults with CP or SB seems to be understudied and it is important to gain insight into useful interventions for mental wellbeing in these adults. There also is a need for accessible and adequate screening, preventive measures and clinical follow-up of health issues.

## Author contributions

JS and SH contributed to the study conception and design, data collection, and interpretation of results. JS performed data analysis and drafted the manuscript. Both authors reviewed the results and approved the final version of the manuscript.
