# Supplementary material for: Impairments and comorbidities in adults with cerebral palsy and spina bifida: a meta-analysis
Source: Front Neurol. 2023 Jul 18;14:1122061. doi: 10.3389/fneur.2023.1122061 (PMC10390785; doi:10.3389/fneur.2023.1122061)
Supplement: Supplementary file 1 [file Data_Sheet_1.docx]

**Supplementary File 1 Search Strategy**

**Pubmed**

(('cerebral palsy' OR 'spina bifida' OR 'duchenne muscular dystrophy' OR 'spinal muscular atrophy') AND adults) AND (pain OR fatigue OR deformities OR 'bladder incontinence' OR 'bowel incontinence' OR anxiety OR depression OR cancer OR 'liver disease' OR 'renal disease' OR copd OR arthritis OR arthrosis OR diabetes OR hypertension OR 'cerebrovascular disease' OR stroke OR 'ischemic cardiac disease' OR asthma OR epilepsy OR 'pressure ulcers' OR bmi)
