# Supplementary material for: Impairments and comorbidities in adults with cerebral palsy and spina bifida: a meta-analysis
Source: Front Neurol. 2023 Jul 18;14:1122061. doi: 10.3389/fneur.2023.1122061 (PMC10390785; doi:10.3389/fneur.2023.1122061)
Supplement: Supplementary file 2 [file Data_Sheet_2.docx]

**Supplementary File 2 Results in adults with CP**

**Comorbidity in adults with CP: Overview of studies, cases and overall proportions**

| **Health issue** | **Number of Studies (refs)** | **Cases in analysis** | **Overall Proportion (95% CI)** |
| --- | --- | --- | --- |
| Anxiety | **4** (6, 47, 69, 94) | 21644 | 20.3 (16.4 – 24.6) |
| Asthma | **5** (38, 47, 84, 89, 114) | 3836 | 24.2 (15.1 – 34.6) |
| Bladder problems | **9** (7, 31, 32, 47, 65, 72, 113, 115, 119) | 25934 | 16.2 (9.2 – 24.8) |
| Bowel problems | **4** (31, 32, 66, 109) | 9450 | 13.8 (0.6 – 39.5) |
| Cancer | **4** (6, 46, 89, 109) | 27711 | 5.9 (2.3 – 11.1) |
| COPD | **5** (38, 46, 89, 113, 115) | 30549 | 7.8 (4.7-11.6) |
| Cardiovascular Disease | **6** (6, 47, 89, 108, 113, 115) | 25450 | 6.2 (4.7 – 7.9) |
| Deformity | **6** (28, 32, 51, 53, 54, 58) | 921 | 44.2 (12.9 – 78.4) |
| Depression | **14** (6, 7, 47, 53, 67, 69, 81, 92, 94, 95, 100, 110, 113, 115) | 64569 | 19.8 (14.9 – 25.2) |
| Diabetes | **18** (6, 7, 10, 38, 46, 58, 80, 84, 89, 94, 95, 114, 108, 109, 112, 113, 115, 118) | 64155 | 9.0 (7.2 – 10.9) |
| Epilepsy | **19** (7, 31, 32, 47, 48, 53, 54, 59, 66, 72, 80, 85, 94, 95, 103, 107, 110, 113, 115) | 53921 | 22.6 (18.5 – 27.1) |
| Fatigue | **11** (7, 31, 52, 55, 56, 64, 74, 79, 93, 100, 110) | 24032 | 36.9 (24.6 – 50.1) |
| Gastroenterological problems | **8** (31, 32, 47, 53, 54, 80, 81, 115) | 18956 | 19.0 (11.9 – 27.3) |
| Heart disease | **10** (32, 38, 46, 53, 84, 94, 95, 114, 118) | 21534 | 8.3 (5.5 – 11.7) |
| Hypertension | **22** (6, 7, 38, 46, 47, 50, 58, 70, 80, 81, 84, 88, 89, 101, 108, 109, 112, 113, 114, 115, 118) | 61239 | 27.3 (23.6 – 31.0) |
| ICD | **6** (6, 32, 89, 108, 113, 115) | 25293 | 5.5 (3.9 – 7.3) |
| Intellectual disability | **12** (7, 32, 47, 51, 54, 67, 72, 95, 103, 107, 113, 115) | 44930 | 37.2 (26.7 – 48.3) |
| Lung disease (other than COPD) | **11** (7, 31, 32, 46, 47, 53, 54, 89, 94, 109, 115) | 40793 | 13.2 (8.9 – 18.2) |
| Obesity | **10** (38, 39, 47, 50, 66, 70, 81, 82, 88, 101) | 1160 | 28.6 (20.5 – 37.6) |
| Osteoarthritis | **12** (6, 7, 38, 49, 77, 84, 94, 106, 110, 114, 115, 113) | 50824 | 13.2 (10.2 – 16.5) |
| Osteoporosis | **7** (6, 38, 47, 77, 106, 113, 115) | 26634 | 10.9 (7.2 – 15.2) |
| Pain | **21** (6, 7, 28, 31, 45, 52, 54, 55, 56, 57, 78, 79, 81, 84, 87, 90, 92, 94, 100, 110) | 33477 | 56.4 (48.8 – 63.8) |
| Renal disease | **7** (6, 7, 46, 109, 112, 115, 118) | 53307 | 3.0 (2.1 – 4.2) |
| Rheumatic disorders | **6** (38, 77, 106, 114, 115, 112) | 21302 | 4.8 (1.5 – 9.9) |
| Stroke | **4** (38, 47, 84, 95) | 3382 | 4.8 (3.4 – 6.5) |
| Vision problems | **5** (31, 32, 52, 54, 72) | 324 | 14.1 (6.6 – 23.8) |
