# Supplementary material for: Impairments and comorbidities in adults with cerebral palsy and spina bifida: a meta-analysis
Source: Front Neurol. 2023 Jul 18;14:1122061. doi: 10.3389/fneur.2023.1122061 (PMC10390785; doi:10.3389/fneur.2023.1122061)
Supplement: Supplementary file 3 [file Data_Sheet_3.docx]

**Appendix C Results in adults with SB**

**Comorbidity in adults with SB: Overview of studies, cases and overall proportions**

| **Health issue** | **Studies** | **Cases in analysis** | **Overall Proportion (95% CI)** |
| --- | --- | --- | --- |
| Diabetes | **4** (62, 75, 96, 105) | 634 | 4.8 (2.8 – 7.3) |
| Obesity | **4** (30, 35, 41, 63) | 544 | 28.9 (21.1 – 37.5) |
| Sleeping problems | **4** (30, 62, 96, 104) | 718 | 30.3 (4.7 – 65.8) |
| Urinary track infection | **5** (44, 73, 97, 102, 105) | 1301 | 29.7 (21.5 – 38.6) |
| Hypertension | **6** (30, 36, 62, 68, 96, 105) | 1041 | 14.6 (6.0 – 26.2) |
| Depression | **6** (29, 40, 62, 91, 104, 105) | 857 | 24.2 (7.8 – 46.0) |
| Pain | **6** (30, 42, 73, 75, 104, 105) | 1346 | 44.1 (27.4 – 61.5) |
| Renal disease | **8** (35, 36, 44, 62, 68, 75, 96, 97, 102) | 1250 | 8.7 (2.0 – 19.9) |
| Bowel incontinence | **8** (30, 34, 35, 68, 76, 97, 102, 104) | 936 | 49.2 (34.5 – 64.0) |
| Epilepsy | **9** (30, 34, 42, 43, 62, 68, 75, 102, 105) | 1126 | 14.1 (8.4 – 21.0) |
| Bladder incontinence | **13** (30, 34, 35, 44, 60, 61, 68, 76, 97, 102, 104, 116, 117) | 3973 | 60.0 (50.5 – 69.2) |
